# An On-Site, Ultra-Sensitive, Quantitative Sensing Method for the Determination of Total Aflatoxin in Peanut and Rice Based on Quantum Dot Nanobeads Strip

**DOI:** 10.3390/toxins9040137

**Published:** 2017-04-13

**Authors:** Suiyan Ouyang, Zhaowei Zhang, Ting He, Peiwu Li, Qi Zhang, Xiaomei Chen, Du Wang, Hui Li, Xiaoqian Tang, Wen Zhang

**Affiliations:** 1 Oil Crops Research Institute of the Chinese Academy of Agricultural Sciences, Wuhan 430062, China; lyracsui@163.com (S.O); htnanobody@163.com (T.H.); zhangqi521@126.com (Q.Z.); chenxiaomei_200870@126.com (X.C.); wang416929@126.com (D.W.); lihui-gf@163.com (H.L.); wtxqtutu@163.com (X.T.); zhangwen@oilcrops.cn (W.Z.); 2 Key Laboratory of Biology and Genetic Improvement of Oil Crops, Ministry of Agriculture, Wuhan 430062, China; 3 Key Laboratory of Detection for Mycotoxins, Ministry of Agriculture, Wuhan 430062, China; 4 Laboratory of Risk Assessment for Oilseeds Products (Wuhan), Ministry of Agriculture, Wuhan 430062, China

**Keywords:** total aflatoxin, quantum dot nanobead, test strip, on-site, peanut and rice, agro-food safety

## Abstract

An on-site, ultra-sensitive, and quantitative sensing method was developed based on quantum dot nanobeads (QDNBs) and a test strip for the determination of total aflatoxins (AFTs) in rice and peanuts. The monoclonal antibody against AFT (mAb_AFT_) was homemade and labeled with QDNB. After the pre-coating of the AFT antigen on the test line (T line), the competitive immunoreactions were conducted between AFT and AFT antigen on the T line with QDNBs-mAb_AFT_. Under optimal conditions, this approach allowed a rapid response towards AFT with a considerable sensitivity of 1.4 pg/mL and 2.9 pg/mL in rice and peanut matrices, respectively. The put-in and put-out durations were within 10 min. The recoveries for AFT in rice and peanut sample matrices were recorded from 86.25% to 118.0%, with relative deviations (RSD) below 12%. The assay was further validated via the comparison between this QDNB strip and the conventional HPLC method using spiked samples. Thus, the design provided a potential alternative for on-site, ultra-sensitive, and quantitative sensing of AFT that could also be expanded to other chemical contaminants for food safety.

## 1. Introduction

Aflatoxins (AFTs) are a group of toxic secondary metabolites, mainly produced by *Aspergillus flavus* and *A. parasiticus*. Among these, aflatoxin B_1_, aflatoxin B_2_, aflatoxin G_1_, aflatoxin G_2_, aflatoxin M_1_, and aflatoxinM_2_ were recognized as human carcinogens (group I) by the International Agency for Research on Cancer as early as 1993 due to their high hepatotoxicity, teratogenicity, mutagenicity, and carcinogenicity [1]. The co-occurrence of total AFTs was found to occur from farm to table and to infect susceptible diversified agro-foods, especially peanuts, rice, maize, and barley, thereby severely threatening agro-food safety and human health. Many countries have set restricted the maximum residue level of AFT in agro-food to 4.0 and 10.0 ng/mL for peanuts in the European Union and Japan, respectively [2].

In order to guarantee the consumption safety of agro-food and ensure human health, the on-site, sensitive, and quantitative sensing of AFT is an indispensable prerequisite, especially in developing countries [3]. The on-site approach could predict an early warning of AFT contamination in agro-food, whereas the sensitive detection could address the issue of trace amounts and inhomogeneous distribution. Currently, only a few reporters were available for the qualitative sensing methods including ELISA, biosensor, and qualitative test strip [4,5,6,7,8,9]. Among these sensing techniques, the test strip can provide a rapid and time/labor-saving sensing method, in a single-step and in a possible on-site manner. Additionally, the test strip can reduce the use of hazardous AFT standard solution and antigens, suggesting a user-friendly format. It can also achieve a sensitive low-cost determination for AFT, thereby guaranteeing potential on-site application. Typically, the gold nanoparticle has been substantiated as an effective reporter for colorimetric detection, allowing qualitative determination for mycotoxin with relatively high concentrations [6,10,11,12]. Furthermore, to meet the requirements of detection of sensitive mycotoxins such as AFT, some quantitative test strip methods have been developed recently using various reporters such as liposome-encapsulated dyes [13], magnetic nanogold microspheres [14],time-resolved fluorescence [15,16,17], up-converting phosphor [18], fluorescent microspheres [19], and quantum dots (QDs) [20,21,22]. Owing to the intrinsically high sensitivity, the fluorescence-based strip could qualify application in food safety [9]. The organic fluorescence could be hampered from extensive application due to its inherent photobleaching, thus lowering the sensitivity. QDs have been proven as one of the optimum reporters. Compared to organic fluorophores, QDs have high quantum yields, exceptional photostability, broad absorption cross-section, size-tunable fluorescence emission, narrow spectral line widths, and excellent stability against photobleaching [23]. However, a single QD could not be sensitive due to the longstanding issue of chemical and colloidal instability in physiological environments after the phase-transfer procedure [24,25]. In order to address these problems, doping or encapsulating the beads as a signal amplification label to gain both good chemical and colloidal stability was effective. For example, Li et al., fabricated a test strip, utilizing quantum dot nanobeads (QDNBs) as probes for prostate specific antigen analysis, finding a LOD (limit of detection) of 12-fold lower than the result obtained by single QDs as fluorescent labels [26]. Although there were similar reporters for ZEA or AFB_1_ determination in agro-products [22,27], there remained great challenge for AFT sensing via the encapsulated or doped QD nanobeads. The reason could be the inevitable matrix effect from complicated agro-product samples, which would significantly arouse the background noise and then reduce the sensitivity. For example, when compared with maize, peanuts, as a typical sample with complex matrix effect, exhibited a obviously lower sensitivity due to their high fat (44–56%) and high protein content (22–30%) [28,29].

To solve the matrix effect, we developed a highly specific anti-AFT monoclonal antibody. Herein, based on the homemade monoclonal antibody, QDNBs as fluorescence reporters were introduced to enhance the sensitivity of a test strip for sensing AFT in peanuts and rice. The sensing performance was validated, including LOD, linear range, recovery, and relative standard deviations (RSDs), using spiked samples. The results of the test strip and the conventional HPLC method were further compared. The QDNBs-based test strip could be extensively applied in the on-site and rapid determination of AFT and other contaminants in agro-food safety.

## 2. Results and Discussion

### 2.1. Principle of the Method

**Figure 1:**
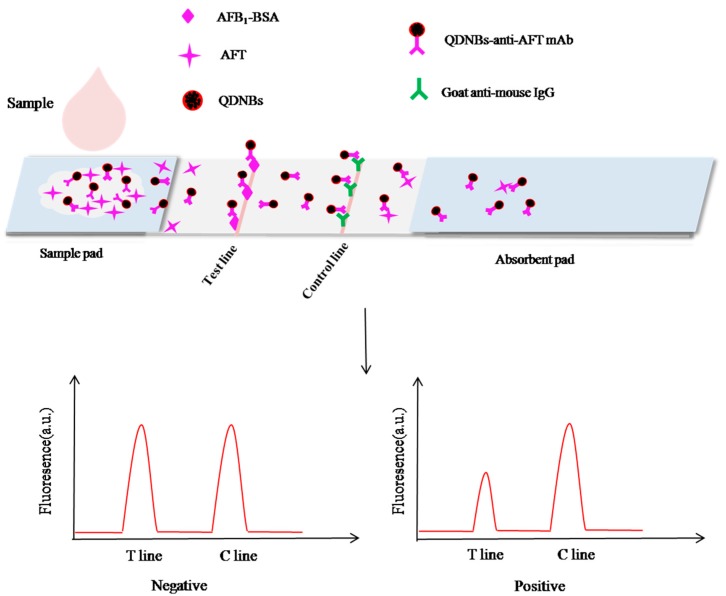
Schematic illustration of a quantum dot nanobeads (QDNBs) strip.

The principle of the QDNB strip was based on the antigen-antibody reaction in an indirect competitive mode when the sample migrated to the end of the strip, as illustrated in Figure 1. The QDNBs demonstrated excellent optical properties compared to QDs and allowed highly sensitive and quantitative detection of AFT. As the liquid sample migrated on the strip, the QDNB-mAb_AFT_ was bound with the AFT or its antigen on the T line (test line). Then the residual QDNB-mAb_AFT_ was captured by the C line (control line). In a contrast assay, the QDNB-mAb_AFT_ were migrated freely to the NC membrane and captured by the C line. After appropriate immunoreaction time, the fluorescence intensities of the T and C lines were detected by a reader. In the presence of AFT, the absence of less QDNB-mAb_AFT_ was captured by the T line, weakening the fluorescence intensity on the T line, and the residual fluorescence probes were captured by the C line. The quantitative analysis of AFT was conducted by recording the fluorescence intensities of the T and C lines. The T/C ratio was used to offset the background and inherent heterogeneity of the strip. It was expected to be inversely proportional to the increasing concentration of AFT in the samples [30]. Standard curves were plotted according to the proportional relationship between the B/B_0_ and analyte in the sample at different concentrations, where B and B_0_ represented T/C with and without the presence of competitive antigen (AFT) in the standard solutions, respectively. In either case, the control line should always be assessed or else the strip will be invalid.

### 2.2. Characterization of QDNB and QDNB-mAb

**Figure 2:**
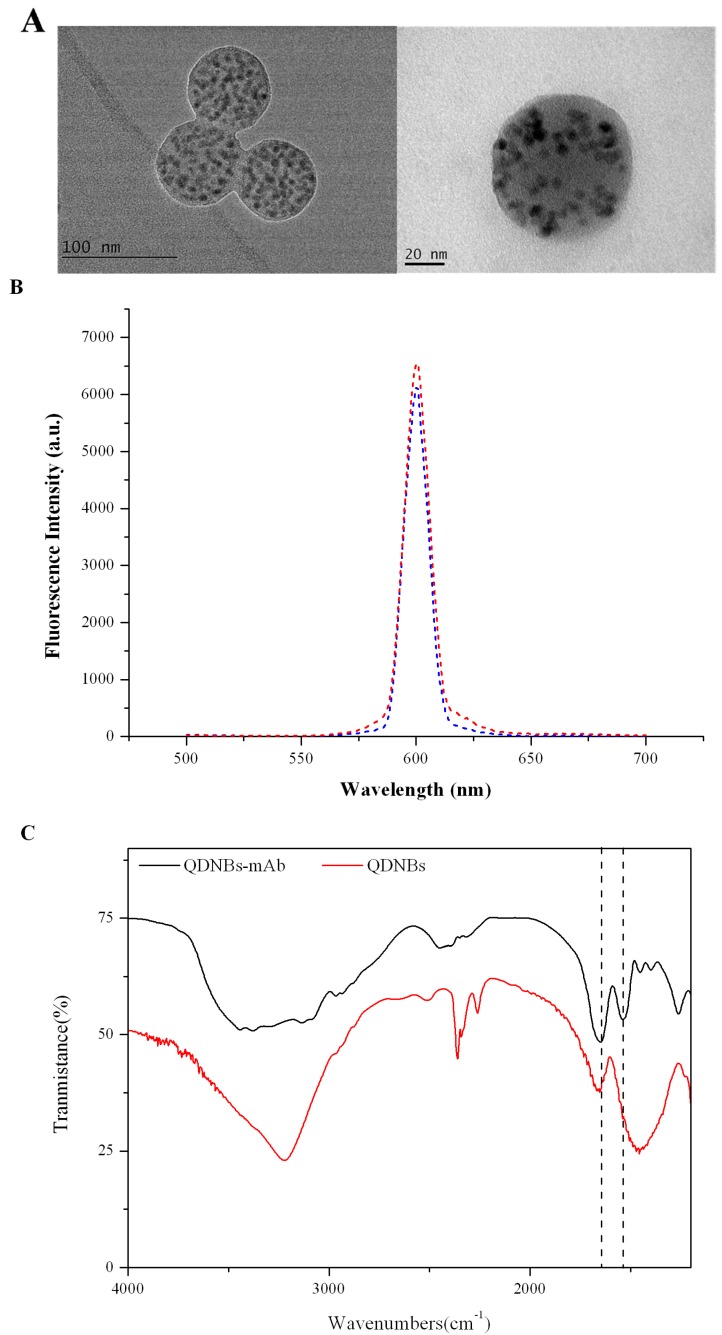
Characterization of QDNBs and QDNBs-monoclonal antibody (mAb). (**A**) TEM images of QDNBs; (**B**) Fluorescence spectra of QDNBs and QDNBs-mAb; (**C**) Characterization of QDNBs and QDNBs- monoclonal antibody against total aflatoxins (mAb_AFT_).

The QDNBs, pre-modified by polystyrene maleic-anhydride copolymer, were used as received. The size and morphology of the prepared QDNBs were characterized via a high-resolution transmission electron microscope (TEM). Figure 2A showed a compact quantum dots-polymer structure, indicating that the numerous, individual, dark dots with a diameter of approximately 6 nm are tightly encapsulated in the polymer matrix. These dark dots are QDs, which can be visibly identified from the polymer matrix, owing to the different electron penetrability between the QDs and polymer matrix [27]. The TEM image indicated that the average diameter of QDNBs is 90 nm.

In order to confirm the binding of the QDNBs and antibody, the fluorescence characteristics of QDNBs were measured before and after coupling with the antibody, respectively. The fluorescence spectrum (Figure 2B) of QDNBs presented a bright and narrow fluorescence peak at 602 nm and suffered from little fluorescence decay after the conjugation of QDNBs and mAb as compared to the unconjuagted QDNBs [31]. A distinct shift in the emission spectra and wavelength of QDNBs-mAb and free QDNBs was not observed, indicating little aggregation during the coupling of the QDNBs and mAb. Therefore, the prepared QDNBs-mAb qualified for the quantitative QDNB strip. Additionally, compared to QDNBs, the FTIR (Fourier-transform infrared) spectra (Figure 2C) of the QDNBs-mAb_AFT_ showed characteristic absorption peaks corresponding to protein amide bands I (1650 cm^−1^) and II (1533 cm^−1^), respectively. The -COOH group on the surface of the QDNBs was successfully covalently bound with the -NH_2_ group in the mAb.

### 2.3. Optimization of Experimental Parameters

As a rapid, quantitative determination method, the performance of the QDNB strip was affected by some parameters such as the properties of the QDNBs-mAb, the volume of the sample solution, and the immunoreaction time. The pH value in the coupling process was optimized. The results showed that the phosphate buffer engendered strong background fluorescence on the junction of the sample pad and nitrocellulose membrane, whereas the borate buffer only slightly enhanced the background noise. When spiked with a series of AFT concentrations, the strip obtained the highest sensitivity in borate solution (pH 7.4). Hence, borate buffer was chosen for coupling process.

The amount of antibody labeled on the QDNBs was also optimized. The fluorescence probes were prepared with different mass ratios of the mAb and QDNBs (0, 45, 60, 80, 100, and 130 μg) in borate buffer. First, the minimal amount of antibody was verified. When the antibody concentration was less than 65 μg/mL, it recorded low fluorescence intensity on the T line and C line. Considering that the test strips for sensing small molecules were based on a competitive immunoreaction, the less usage of the mAb, the higher sensitivity that could be achieved. The results showed that 100 μg of labeled antibody displayed the highest sensitivity when spiked with a standard solution with a gradient of AFT concentration. Therefore, the optimized antibody concentration was 100 μg/mL of the reaction solution.

**Figure 3:**
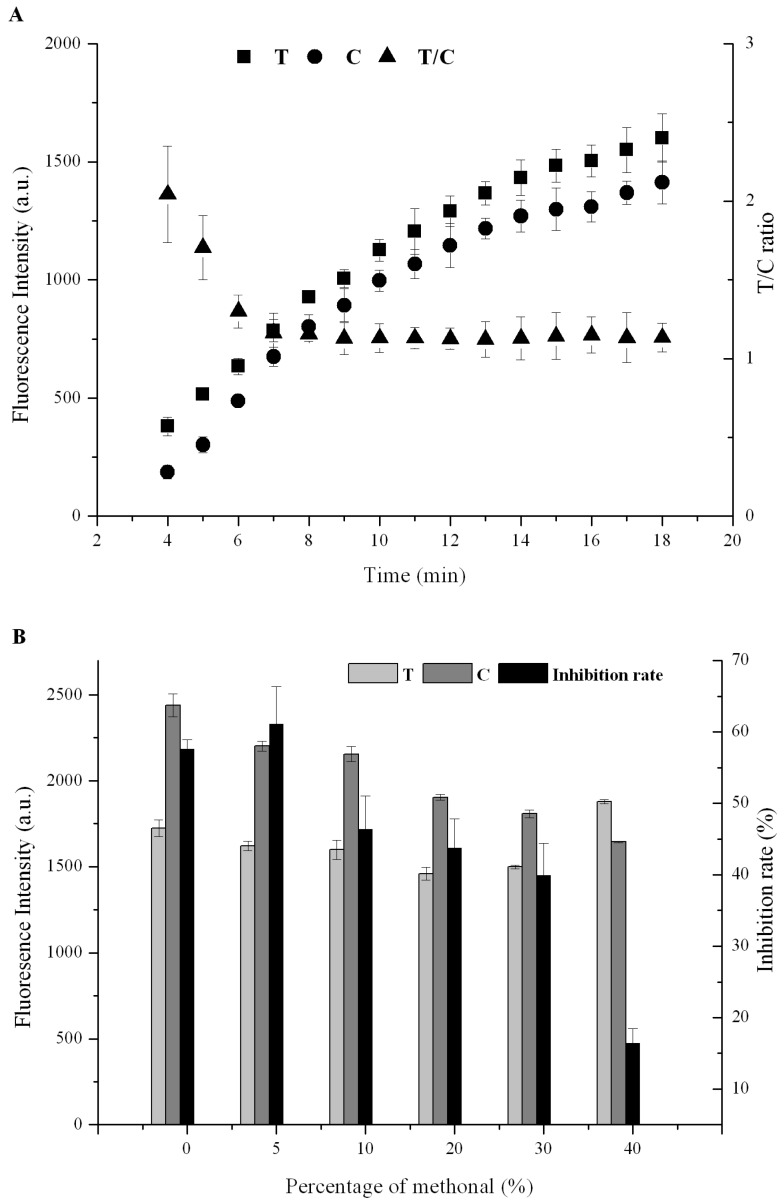
(**A**) Dynamic change of the test to control (T/C) ratio over immunoreaction time; (**B**) Effect of percentage of methanol on test (T), control (C), and inhibition rate.

Furthermore, the loading sample volume (60, 80, 100, 120,150 mL) was investigated, indicating that, in the case of less than 80 mL, the QDNBs could not react thoroughly with the antigen and secondary antibody during the lateral flow. When the sample volume was over 100 mL, the strip gained favorable luminosity and desirable assay performance. Thus, considering the sample consumption and sensitivity, the optimal sample volume was set at 100 mL. A number of fluorescence probes affected the fluorescence response of the QDNBs strip, as the intensity depends on the amount captured on the T line. The inadequate QDNB-mAb could not satisfy the AFT sensing with a relatively high concentration, which may be attributed to the inability of the antigen on the T line to totally capture the mAb, resulting in a narrow dynamic range. However, a large amount of QDNBs-mAb would lower the sensitivity due to high background noise from excessive fluorescence of QDNBs. In order to optimize the amount of QDNBs-mAb, the prepared QDNBs-mAb were mixed in a series of concentration ratios to perform AFT sensing. Thus, an optimized QDNBs-mAb consumption of 2 μL in a 100 mL sample volume was determined. To estimate the reaction time, the assay was carried out under different immunoreaction durations. Figure 3A shows the relationship between T, C, the T/C ratio, and different immunoreaction times. It was found that with a prolonged reaction time, T and C increased simultaneously and the T/C ratio remained constant after 10 min. This suggested that the immunoreaction could be fully completed during the flow migration along the NC membrane within 10 min; hence the optimal reaction time was set at 10 min.

In order to ensure recovery, the solution containing 70% methanol was used to extract the hydrophobic AFT from the peanut and rice samples. It is well established that the high concentration of methanol during immunosensing would cause mAb denaturation concurrently with the lateral flow by altering the mAb configuration and decreasing the bioactivity of the QDNBs-mAb conjugates. To address this issue, the extraction buffer was diluted before lateral flow on the strip. The results (Figure 3B) indicated that less than 30% methanol in the sample loading solution exhibited negligible effects on the fluorescence intensity of the T line, C line, and the T/C ratio. When the methanol concentration was less than 5%, the immunochromatographic assay (ICA) test gained the highest inhibition rate, resulting in a desirable sensitivity. Considering the ultra-sensitivity of the QDNBs strip, the rice and peanut extracts were then diluted with running buffer to a final methanol concentration of 5% for subsequent experiments.

### 2.4. Evaluation of the QDNBs Strip

**Figure 4:**
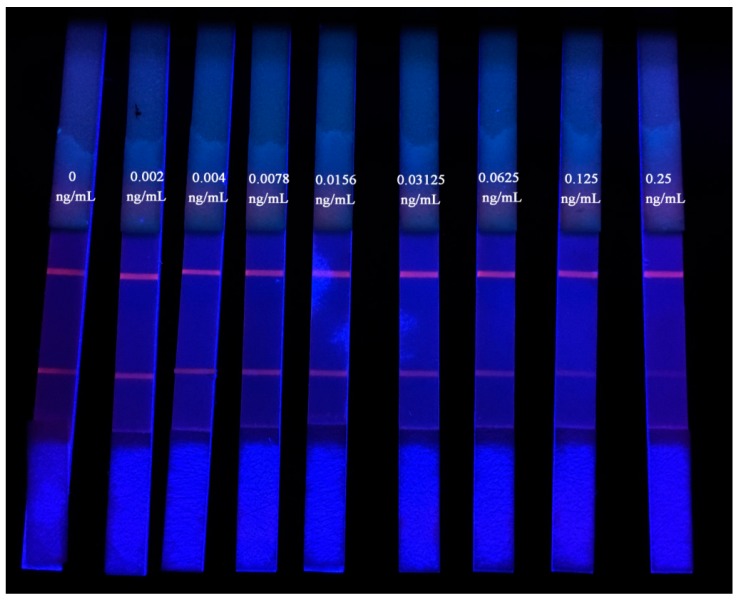
Gradient concentration of the aflatoxin (AFT)-spiked buffer under UV light.

**Figure 5:**
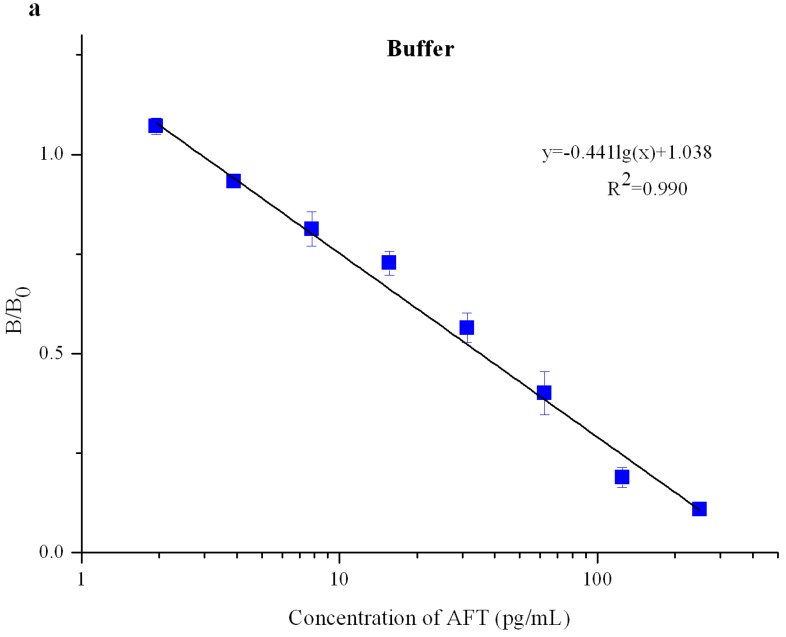

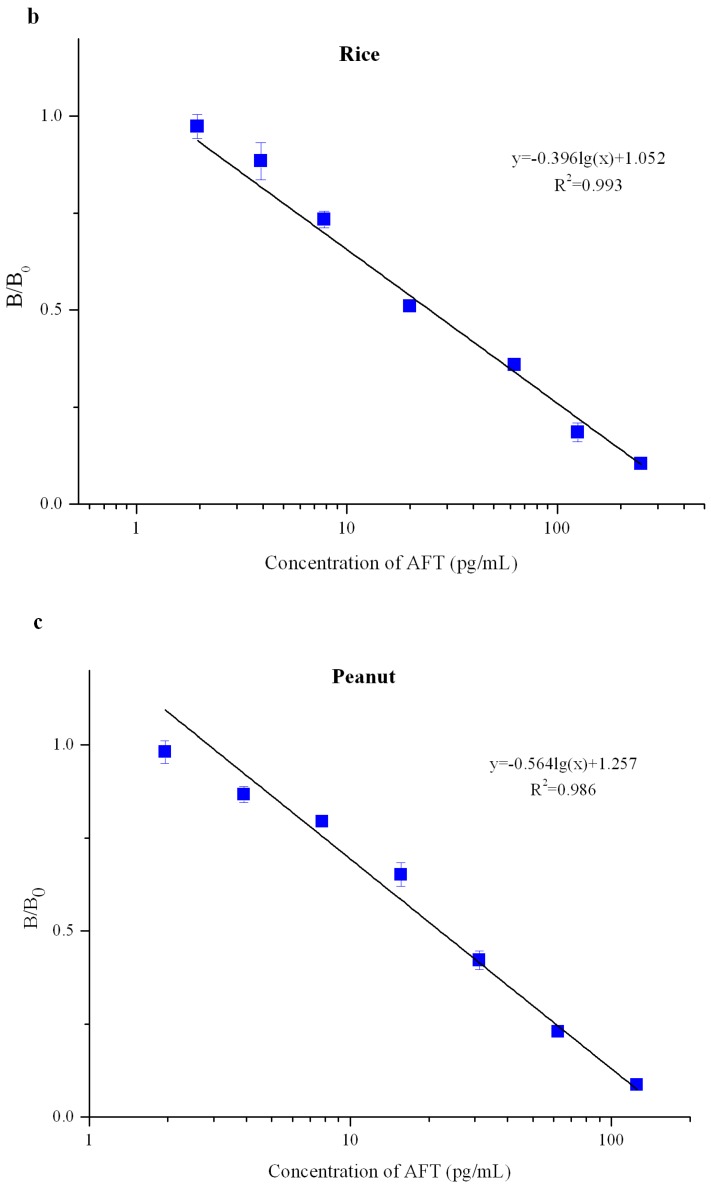
Standard curves for AFT quantitative detection in buffer (**a**); rice (**b**); and peanut (**c**) matrices.

The QDNBs strip was evaluated under optimal experimental conditions. A quantitative calibration curve was drawn according to the scanned peak intensity ratio of B/B_0_ against the log of AFT concentration, where B and B_0_ represent the T and C lines, respectively, with and without the presence of competitive antigen (AFT) in the standard solutions (Figure 4 and Figure 5). After five repetitions of the experiments, the LODs were calculated as the mean value by the calibration curve using 21 blank samples. The proposed QDNBs-based ICA strip exhibited linearity over 2–250 pg/mL with a LOD of 1.2 pg/mL in the buffer. The calibration equation was determined to be *y* = −0.441log(x) + 1.038 with a reliable correlation coefficient (*R*^2^ = 0.990), where y represents the value of B/B_0_ and *x* was the log of the AFT concentration. Due to the matrices effects, the LODs obtained in AFT-spiked rice and peanuts were 1.4 pg/mL and 2.9 pg/mL, respectively. Compared to the result in the buffer, the narrower dynamic range of 2–125 pg/mL was obtained in spiked peanut extracts while the linear range for rice matrices was not a detriment.The RSDs for AFT analysis in the working range of the calibrations curves were 2.0–12.9% in rice samples and 1.3–11.7% for peanut samples.

**Figure 6:**
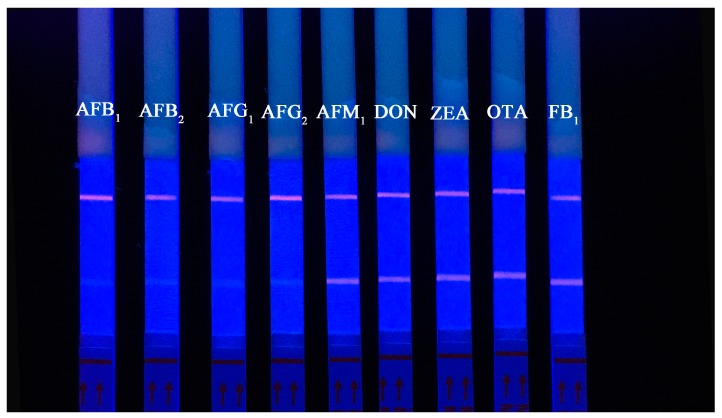
Cross-reactivity results with spiked AFM_1_, DON, ZEA, OTA, and FB_1_ samples (0.5 ng/mL).

To estimate the specificity of the QDNBs-based ICA strip, the cross-reactivity between AFT and other mycotoxins (AFM_1_, DON, ZEA, OTA, and FB_1_) was assessed via a fixed concentration of 0.5 ng/mL (triplicate). In Figure 6, the fluorescence intensity in the T line decreased only slightly, even with a high concentration of other mycotoxins. Therefore, the cross-reactivity between AFT and other toxins was negligible, and AFT could be specificallydetected via the QDNBs strip.

Recovery was investigated by peanut and rice samples spiked with a series of AFT concentrations. Table 1 demonstrated that the recoveries ranged from 86.25% to 118.0% and 91.25% to 106.25% for rice and peanuts, respectively. The recovery in peanuts was narrower than the one in rice, likely due to the different matrix effect. The results found that all the recovery data could satisfy the application criteria of the QDNB strip, according to the regulations in the EU, USA, and China.

The inter-batch and intra-batch variabilities were assessed by repeated experiments.The spiked rice and peanut extracts were used for the low, medium, and high levels of AFT concentrations. Three spiked rice extracts with 10, 50, and 100 pg/mL AFT and three spiked peanut extracts with 10, 50, and 80 pg/mL AFT were introduced to test the inter-assay and intra-assay with excellent reproducibility (Table 2).The variations of the intra-assay and inter-assay were 4.2–10.3% and 6.3–11.2%, respectively. This indicated that the QDNB strip was a practical tool for quantitative sensing of AFT.

A total of five rice and five peanut samples were used to compare the sensing results from the QDNB strip and the standard HPLC method. As listed in Table 3, the detection results of the QDNBs-based ICA for all negative or positive samples were in good agreement with the reference HPLC method; the coincidence rate was 100%.

Immunochromatographic assays have been widely employed in the analysis of AFT (mainly only for AFB_1_). Remarkably, Ren et al., demonstrated a quantum dots beads-based ICA with an extremely sensitive performance for AFB_1_ detection with a LOD of 0.42 pg/mL [27]. However, very few have been used for total AFTs. For example, Zhang et al., fabricated a qualitative nanogold probe-based ICA test for total AFT with a desirable visual LOD [6]. Tayebi et al. determined the total aflatoxins using cysteamine-capped CdS quantum dots as a fluorescence probe based on Langmuir Adsorption model with an LOD of 0.05 ng/mL [8]. Compared to the current studies on ICA analysis for AFT (Table 4), the present approach revealed a more sensitive detection and broader working range. All the results indicated that the QDNBs-based ICA exhibited an ultra-sensitive and highly reproducible performance, thereby demonstrating potential in the point-of-care application.

## 3. Conclusions

The QDNBs, as alternative fluorescence reporters, were integrated on the lateral flow test strip platform, allowing an on-site, ultra-sensitive, and quantitative evaluation method for the determination of AFTs in peanut and rice within 10 min. The hommade mAb was labeled on QDNBs. This approach allowed a rapid response towards AFT with a considerable sensitivity of 1.4 pg/mL and 2.9 pg/mL in rice and peanuts, respectively. The recoveries for AFT in rice and peanut sample matrices were recorded from 86.25% to 118.0% with RSD below 12%. These results provided a potential alternative design for on-site, ultra-sensitive, and rapid AFT quantitation, as well as the expansion of the technique to other chemical contaminants with respect to food safety.

## 4. Experiments

### 4.1. Materials and Instruments

Anti-AFT mAb was generated in our laboratory. Bovine serum albumin (BSA), *N*-(3-dimethyla-minopropyl-*N*′-ethylcarbodiimide hydrochloride (EDC), *N*-hydroxysuccinimide (NHS), and AFB_1_-BSA conjugate were purchased from Sigma-Aldrich (St. Louis, MO, USA). Goat anti-mouse immunoglobulin was acquired from Wuhan Boster Biological Technology, Ltd. (Wuhan, China). QDNBs, pre-modified by polystyrene maleic-anhydride copolymer, were provided by Shanghai Kundao Biotech Co., Ltd. (Shanghai, China). Nitrocellulose membrane, sample pad, absorbent pad, and plastic adhesive card were purchased from Millipore Co. (Bedford, MA, USA). All other inorganic chemicals and organic solvents were of analytical reagent grade unless stated otherwise. Ultrapure water (18.2 MΩ·cm) was obtained with a Millipore Milli-Q system and used for the preparation of all the solutions.

The TEM images were acquired on an FEI Tecnai G2 20 electron microscope. The FTIR spectra images were obtained on TENSOR II FTIR Spectrometer, Bruker (Karlsruhe, Germany). The photoluminescence spectrum of the QDNBs was recorded by a fluorescence spectrophotometer (Hitachi F-4500, Tokyo, Japan). An XYZ3050 Dispensing Platform, CM4000 Guillotine Cutter, and LM4000 Batch Laminator (BioDot, Irvine, CA, USA) were used to prepare the test strips. A portable strip reader was provided by Microdetection Co., Ltd. (Nanjing, China). The high-speed freezing centrifuge (CF16RX) was from Hitachi (Tokyo, Japan). The results were substantiated with an Agilent 1200 HPLC system (Agilent Tech, Santa Clara, CA, USA).

### 4.2. Preparation of the QDNB-mAb

QDNBs-mAb conjugates were prepared according to the protocol for the QDNBs antibody conjugation. Briefly, 3 mL QDNBs dispersed in 0.05 M MES (2-[*N*-Morpholino]ethanesulfonicacid, pH 6.0)were activated with 12 mg EDC and 18 mg NHS at room temperature for 15 min. Subsequently, the COOH-activated QDNBs were separated by centrifugation at 13,300 rpm for 10 min, and the precipitates were resuspended in 3 mL borate buffer (0.01 M, pH 7.4) to react with the antibody for 4 h at room temperature with gentle agitation to obtain the QDNBs-mAb conjugates. Then, the mixture was centrifugated again at 13,300 rpm for 10 min, and the resulting precipitates were solubilized in 5 mL of borate buffer(0.01 M, pH 7.4) containing 1% BSA, gently agitated to block the unsaturated sites for an additional 1 h, and stored at 4 °C until further usage.

### 4.3. Fabrication of the QDNBStrip

QDNBs strips were composed of a sample application pad, nitrocellulose membrane, absorption pad, and a backing card. The AFB_1_-BSA conjugate and goat anti-mouse IgG were used for the T line and C line, respectively, and then coated on the nitrocellulose membrane using the BioDot XYZ3050 Platform at an adequate jetting rate. The sample pads were treated with blocking buffers (0.01 M PBS, containing 0.5% (*w*/*v*) BSA, 0.5% (*v*/*v*) Tween-20, 0.5% (*w*/*v*) PVP-K30, 0.25% (*w*/*v*) EDTA, 0.05% (*w*/*v*) sodium azide) and dried at 37 °C overnight. The absorption pad was utilized without treatment. The coated membrane, sample pad, and absorbent pad were laminated and pasted to a plastic scale-board. Then, the assembled scale-board was cut laterally with a guillotine cutter (CM 4000) and divided into 4 mm × 60 mm strips. Finally, the strips were stored in a self-sealing plastic bag with desiccant at 4 °C.

### 4.4. Determination Procedure via the QDNB Strip

To enhance the sensitivity and reliability, the optimization of the QDNBs-based test strip was conducted as described previously. The following parameters were optimized: the pH value, the amount of mAb labeled on the QDNBs, the amount of QDNBs, the sample volume, and the reaction time. For the assay, the sample (AFT standard or cereal samples) was mixed with a specific amount of QDNBs-mAb conjugates with running buffer (distilled water containing 1% sucrose and 1% PVP-K30) in a 96-well plate (the final volume was 100 μL) in duplicate (*N* = 5). Then the mixture was loaded onto the sample pad, allowing all liquid to be absorbed and to migrate along the strip. After 10 min, the fluorescence intensities of the T line and C line on the lateral flow test strip were acquired with a fluorescence detector at an emission wavelength of 600 nm. Quantitative analysis was performed by recording the fluorescence intensity of T line and C line as well as the T/C ratio. The standard curve was established by plotting the B/B_0_ against the log of AFT concentration, where B and B_0_ represented the T/C ratio with and without the presence of competitive antigen (AFT), respectively, in the standard solutions. The standard AFT solutions were prepared by spiked stock AFT solution and diluted in a gradient.

### 4.5. Evaluation of the QDNB Strip

The spiked peanut and rice samples, with AFT concentrations including 0, 0.002, 0.004, 0.0078, 0.0156, 0.03125, 0.0625, 0.125, and 0.25 ng/mL, were employed for the evaluation of the QDNB test strip. The standard curve, LOD, linear range, and specificity were estimated as described in our previous report [17]. For standard curves and LOD on a QDNBs-based ICA strip, the fluorescence intensities of the T and C lines were used for sensitive and quantitative detection. The linear range started from 3-fold above the LOD and ended at the cut-off concentration where the color faded or the linear range exceeded. A standard recovery procedure was used for AFT recovery, utilizing a series of AFT-spiked peanut and rice samples. The specificity was investigated via appropriate amounts of other mycotoxins (0.5 ng/mL) by cross-reactions of the QDNB-test strip with AFM_1_, DON, ZEA, OTA, and FB_1_. The inter and intra-batch precision with AFT-spiked samples was studied using a QDNB-test strip, and the assay was repeated five times.

### 4.6. Sample Treatment for the QDNB Strip

The sample was finely ground with a laboratory mill, and 10 g was placed in a 50 mL centrifuge tube. 30 mL methanol-water (70:30, *v*/*v*) was vigorously mixed with the powder for overnight extraction. Subsequently, the supernatant was transferred to a 1.5 mL tube and diluted to the final methanol concentration of 5% methanol. The resulting solution was used in the QDNB-test strip.

### 4.7. Sample Treatment for HPLC Analysis

The sample was extracted as described above, followed by purification on an immunoaffinity column. 1 mL sample in methanol was injected into the HPLC equipped with fluorescence detector (λ_ex_ = 365 nm, λ_em_ = 440 nm). The analytical column comprised of an Alltima C18 (150 × 4.6, particle size 5 µm, Alltech, Grace, IL, USA). The mobile phase consisted of a mixture of HPLC grade methanol-water (55:45), eluted at a flow rate of 1.0 mL/min. The temperature of the column was 25 °C, and the injection volume of the suspended sample was 10 µL.

## Figures and Tables

**Table 1:** Recoveries in rice and peanut samples.

Samples	Concentrations pg/mL		Recovery Rates (%)	RSD (%) (*n* = 5)
	Spiked	Detected		
Rice 1	8	6.9	86.25	10.3
	50	59	118.0	8.5
	300	Positive ^a^	- ^b^	-
Rice 2	8	8.4	105.0	9.4
	50	47	94.0	5.8
	300	Positive	-	-
Rice 3	8	7.7	96.25	10.2
	50	51	102.0	6.9
	300	Positive	-	-
Peanut 1	8	7.3	91.25	11.8
	50	46	92	10.3
	300	Positive	-	-
Peanut 2	8	8.5	106.25	4.6
	50	48	96	6.1
Peanut 3	300	Positive	-	-
	8	9.1	106.25	5.7
	50	52	104	5.3
	300	Positive	-	-

^a^ Without T line. ^b^ Not calculated.

**Table 2:** Intra- and inter-batch evaluation.

Samples	Spiked AFT pg/mL	Intra-Batch		Inter-Batch ^a^	
		Mean ^b^	CV%	Mean ^b^	CV%
Rice	10	9.7	5.6	9.8	7.2
Rice	50	59	10.3	57	11.2
Rice	100	94	8.6	98	9.4
Peanut	10	13	4.2	10	6.3
Peanut	50	46	7.5	47	8.7
Peanut	80	73	4.9	70	6.5

^a^ ICA tests were performed every 3 d for 15 d. ^b^ Mean value of 5 replicates at each spiked concentration.

**Table 3:** Comparison of QDNBs strip and the HPLC method.

Samples	AFT Result by HPLC ^a^ ng/mL	QDNBs Based ICA ng/mL
Rice 1#	ND ^b^	ND
Rice 2#	0.53	0.49
Rice 3#	10.52	9.46
Rice 4#	ND	ND
Rice 5#	0.97	0.79
Peanut 1#	52.10	60.28
Peanut 2#	12.35	11.87
Peanut 3#	25.20	24.0
Peanut 4#	156.41	142.49
Peanut 5#	ND	ND

^a^ Average value of three duplicates. ^b^ Not detected.

**Table 4:** Comparison of QDNB strip and other reports on aflatoxin sensing.

Method	Aflatoxin	Samples	LOD	Linear Range	Reference
QDNBs based ICA	AFTs	Rice	1.4 pg/mL	2–250 pg/mL	This work
		Peanut	2.2 pg/mL	2–125 pg/mL	
TRFIA based ICA	AFTs	Feed	0.16 ng/mL	0.48–30.0 ng/mL	[16]
Colloidal gold based ICA	AFTs	Peanut	Visual LOD of 0.03, 0.06, 0.12, 0.25 ng/mL for AFB_1_, B_2_ G_1_ G_2_ respectively		[6]
Langmuir adsorption model	AFTs	-	0.05 ng/mL	2.4–48 ng/mL	[8]
QBs based ICA	AFB_1_	Maize	0.42 pg/mL	5–60 pg/mL	[27]
Magnetic Mesoporous Silica Nanocontainers for Fluorescence Immunoassay	AFB_1_	Peanut	8pg/mL	0.01–5 ng/mL	[32]
TRFIA based ICA	AFB_1_	Soybean sauce	0.1 ng/mL	0.3–10 ng/mL	[15]
Colloidal gold based ICA	AFB_1_	Plant oil	Visual LOD of 1.5 ng/mL	0.0125–2 ng/mL	[10]
